# J-shaped relationship between creatinine levels and the risk of three major adverse events in patients after percutaneous coronary intervention

**DOI:** 10.3389/fendo.2026.1832279

**Published:** 2026-04-20

**Authors:** Xiang Zhu, Xiaqin Zha, Jiali Su, Yuanan Lu, Chao Yu, Lei Wu

**Affiliations:** 1 School of Public Health, Jiangxi Provincial Key Laboratory of Disease Prevention and Public Health, Jiangxi Medical College, Nanchang University, Nanchang, China; 2 Office of Public Health Studies, University of Hawaii at Manoa, Honolulu, HI, United States; 3 Department of Cardiovascular Medicine, Second Affiliated Hospital of Nanchang University, Nanchang University, Nanchang, China

**Keywords:** acute myocardial infarction, adverse events, creatinine, percutaneous coronary intervention, restricted cubic spline

## Abstract

**Background and aims:**

To evaluate the correlation between creatinine (Cre) level and the risk of three kinds of adverse events in patients after percutaneous coronary intervention (PCI), and to clarify its potential correlation threshold and independent predictive value.

**Methods and results:**

This investigation was designed as a retrospective cohort analysis, encompassing 3, 878 individuals diagnosed with acute myocardial infarction who received PCI between January 2018 and December 2020. The primary outcomes were three types of adverse events that occurred post-procedure. To explore the relationship between Cre levels and the outcome measures, a restricted cubic spline model was employed, while the Cox proportional hazards regression model assessed the independent predictive significance. There were 996 instances (25.7%) of adverse events reported. The evaluation using a restricted cubic spline model revealed a notable J-shaped relationship between creatinine levels and the likelihood of three types of adverse events following PCI (nonlinear *P* < 0.05), with the inflection point identified at 110 μmol/L. When considering Cre=110 μmol/L as the baseline, patients with Cre levels below this threshold (low value group) exhibited a 15.5% increase in MACE risk (95% CI: 1.006-1.327, *P* = 0.0416), a 15.6% increase in NACE risk (95% CI: 1.004-1.330, *P* = 0.0436) and a 16.1% increase in MACCE risk (95% CI: 1.015-1.329, *P* = 0.0301).

**Conclusion:**

There is a J-shaped correlation between Cre level and the risk of three kinds of adverse events in patients after PCI, and the inflection point of 110 μmol/L can be used as the key threshold for clinical risk stratification and individualized intervention.

## Introduction

Acute myocardial infarction (AMI) represents a prevalent and serious emergency associated with coronary heart disease, placing significant strain on healthcare systems worldwide (1). Individuals experiencing AMI are at a heightened risk for negative health outcomes, with around 605, 000 new instances and 200, 000 recurrent cases reported each year in the United States. The likelihood of experiencing cardiac arrest or sudden cardiac death within three months of the event is approximately 0.29% (2). In this scenario, the incidence of major adverse cardiovascular events (MACE) among AMI patients following percutaneous coronary intervention (PCI) is relatively frequent, as the tissue damage linked to PCI can result in complications such as inadequate stent expansion, bleeding, myocardial injury, and restenosis, all of which are significant factors contributing to MACE (3, 4). Importantly, even after undergoing PCI, patients remain at risk for life-threatening conditions due to insufficient MACE prevention (5, 6). Early identification of AMI patients who are at an elevated risk for MACE shortly after PCI is vital for implementing timely interventions, enhancing patient communication, and refining treatment strategies, all of which are critical for reducing risks and improving patient outcomes.

Creatinine (Cre), a metabolic byproduct of muscle metabolism excreted primarily by the kidneys, is a widely used clinical marker for evaluating renal function. It is often found at abnormal levels in patients who have undergone PCI (7). Research has indicated that a notable rise in creatinine levels, surpassing a specific limit, acts as an independent risk factor for acute kidney injury (AKI) in critically ill individuals (8, 9). However, it remains uncertain whether baseline creatinine levels in creatinine post-PCI are more closely linked to long-term MACE in AMI patients undergoing PCI, and the optimal creatinine threshold for predicting post-PCI adverse outcomes has not been uniformly defined. This study is designed to explore the relationship between baseline creatinine levels as well as long-term MACE among AMI patients undergoing PCI.

## Methods

### Study design and participants

A total of 4, 541 patients diagnosed with AMI who received PCI were retrospectively analyzed from three medical facilities, including the Second Affiliated Hospital of Nanchang University, during the period from January 1, 2018, to December 31, 2020. The classification of the disease was based on the fourth universal definition of myocardial infarction (10). Individuals with prior PCI or coronary artery bypass grafting, those experiencing recent or ongoing bleeding, or those lacking follow-up information were excluded from the study. This investigation adheres to the principles outlined in the Helsinki Declaration and has received approval from the Ethics Review Committee at the Second Affiliated Hospital of Nanchang University (approval number: No.Review[2017]No.(098)). Informed consent was obtained from all participants involved. The inclusion criteria: ① Age ≥ 18 years old; ② Clinically confirmed AMI; ③Underwent PCI treatment in the hospital and had at least one stent implanted. The exclusion criteria are missing follow-up or lack of complete follow-up information. After stepwise screening, a total of 3, 878 eligible patients were finally included in the statistical analysis. The detailed patient screening, inclusion and exclusion process is presented in **Supplementary Figure 1**.

### Data collection

All data were collected by trained clinical researchers using a unified, prespecified case report form (CRF) from the electronic medical record system of each participating center, with standardized data collection and dual-person quality control protocols to ensure data consistency and accuracy. In this study, demographic characteristics (age, gender, BMI), common comorbidity (hypertension, hyperlipidemia, diabetes), AMI type (ST/NST), Cre and other data were collected before PCI. The study adhered to the STROBE guidelines for observational research (11).

### Endpoints

The main outcomes assessed were three categories of negative events: (1) MACE (12), which primarily encompassed cardiac fatalities, heart attacks, chest pain, heart failure, revascularization procedures, severe arrhythmias, and stent thrombosis, among others; (2) Net adverse clinical events (NACE) (13), which included fatalities, heart attacks, strokes, or significant bleeding from any cause; and (3) Major adverse cardiovascular and cerebrovascular events (MACCE) (13), which mainly involved cardiac death, heart attacks, angina, heart failure, revascularization, severe arrhythmias, stent thrombosis, and strokes. During the follow-up phase, two specialists in cardiovascular and cerebrovascular diseases evaluated both inpatient and outpatient medical records, along with conducting telephone interviews, to verify the occurrence of adverse events through expert agreement.

### Statistical analysis

Statistical evaluations were performed utilizing R software (version 4.4.2), with a significance threshold established at below 0.1 for both tails. The variation of Cre across various genders and types of AMI is illustrated through a violin plot. T-test/Mann-Whitney U-test was used for continuous variables, and Chi-square test was used for classified variables to compare the baseline characteristics among adverse event groups. The overall missing rate of all core variables included in the analysis was 0.13%, which was below the 5% acceptable threshold for complete case analysis. The primary analysis was performed using the complete case analysis method, excluding cases with missing values in any core variable. The variance inflation factor (VIF) was calculated for all variables included in the multivariable model to assess multicollinearity. A VIF value < 5 was defined as no significant multicollinearity. There was no multicollinearity among the features (**Supplementary Figure 1**).

The Cox regression analysis was employed to examine how varying levels of Cre correlate with mortality rates over a one-year period. The impact is measured using hazard ratios (HRs) along with a 95% confidence interval (CI). Furthermore, the association between creatinine levels and the likelihood of negative outcomes is elucidated through the use of a restricted cubic spline (RCS) curve.

A multivariate Cox regression and restricted cubic spline analysis were performed, taking into account various baseline factors such as demographic details (age, gender), AMI type, and medical history (hypertension, hyperlipoidemia, diabetes). Furthermore, a subgroup analysis was conducted for a more in-depth examination of the data. The relationship between creatinine levels and three types of adverse events was analyzed in a hierarchical manner based on gender.

## Results

### Patient characteristics

**Figure 1:**
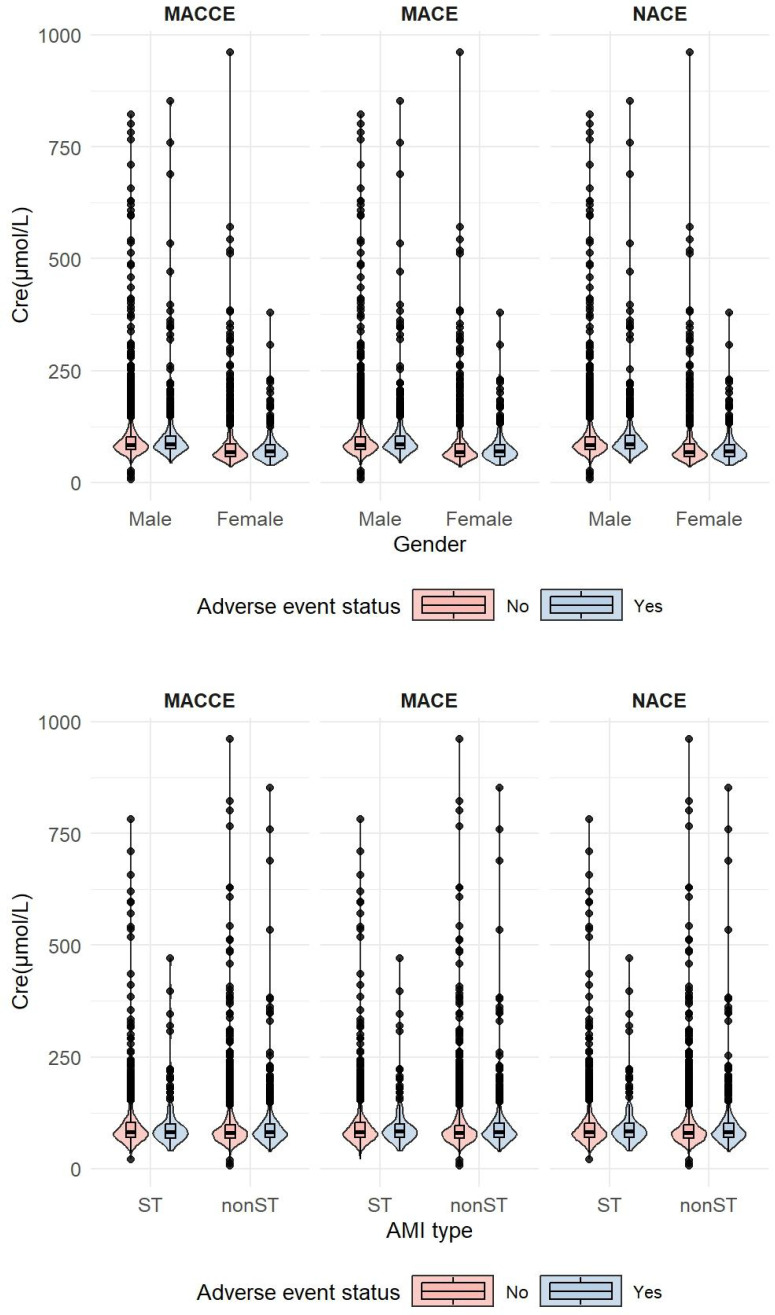
Distribution of Cre in different adverse event types and genders/AMI types.

A total of 4, 541 patients who underwent PCI were included in our study, while 663 patients were not considered due to lack of Cre data (**Supplementary Figure 1**). We analyzed the adverse events related to Cre among these 3, 878 individuals, taking into account gender and AMI type, and created a violin plot to illustrate our findings. The analysis indicated that the distribution of Cre across the various groups was not significantly different (*P* > 0.05) (**Figure 1**). In addition, the results of univariate analysis showed that there was no statistically significant difference in most baseline characteristics among the adverse event groups (*P* > 0.05) (**Supplementary Table 1**).

### Adverse events

Subsequently, we performed a multivariate Cox analysis on three types of adverse events sequentially. The results indicated that AMI type was a significant risk factor for MACE (*HR*_MACE_ = 1.521, *P*_MACE_ < 0.0001), NACE (*HR*_NACE_ = 1.523, *P*_NACE_ *<*0.0001), and MACCE (*HR*_MACCE_ = 1.505, *P*_MACCE_ *<*0.0001). Additionally, diabetes was identified as a risk factor for MACE (*HR*_MACE_ = 1.137, *P*_MACE_ = 0.0693), NACE (*HR*_NACE_ = 1.151, *P*_NACE_ = 0.0492), and MACCE (*HR*_MACCE_ = 1.134, *P*_MACCE_ = 0.0696) (**Table 1**).

**Table 1:** Multivariate Cox analysis of Cre and baseline characteristics in adverse events.

Features	MACE		NACE		MACCE	
	*β*_MACE_	*P*_MACE_	*β*_NACE_	*P*_NACE_	*β*_MACCE_	*P*_MACCE_
Gender	-0.1176	0.1513	-0.1255	0.1315	-0.1229	0.1254
Age	0.0039	0.2263	0.0050	0.1209	0.0048	0.1231
BMI	0.0092	0.4719	0.0166	0.1997	0.0086	0.4894
AMI type	0.4195	<0.0001	0.4206	<0.0001	0.4088	<0.0001
Hypertension	0.1103	0.1082	0.1109	0.1108	0.1242	0.0642
Hyperlipoidemia	0.0095	0.8967	-0.0230	0.7579	-0.0104	0.8855
Diabetes	0.1287	0.0693	0.1408	0.0492	0.1257	0.0696
Smoking	0.0030	0.9728	0.0209	0.8147	0.0006	0.9945
Drinking	-0.0304	0.7075	-0.0053	0.9482	-0.0045	0.9547
Cre	-0.0001	0.8776	-0.0001	0.8333	-0.0003	0.5823

### Nonlinear analysis

**Figure 2:**
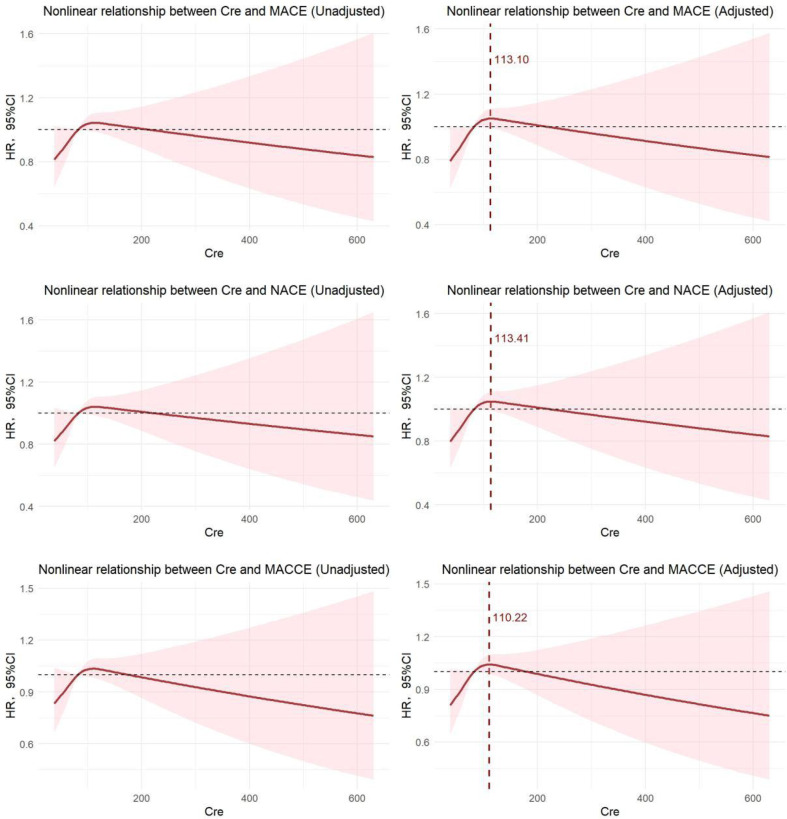
Nonlinear relationship between Cre and adverse events (before and after adjusting AMI type and diabetes).

Analysis using restrictive cubic splines, without accounting for additional variables, indicated a notable nonlinear association between creatinine levels and three types of adverse events (MACE: *P*_total_ = 0.2089, *P*_non-linear_ = 0.0794; NACE: *P*_total_ = 0.2508, *P*_non-linear_ = 0.1005; MACCE: *P*_total_ = 0.2527, *P*_non-linear_ = 0.0974). Even after controlling for factors such as AMI type and diabetes, the nonlinear relationship between creatinine and the risk of these adverse events persisted (MACE: *P*_total_ < 0.0001, *P*_non-linear_ = 0.0464; NACE: *P*_total_ < 0.0001, *P*_non-linear_ = 0.0596; MACCE: *P*_total_ < 0.0001, *P*_non-linear_ = 0.0579). Additionally, we examined the inflection points of the three cubic spline curves, revealing that these points are clustered around a creatinine level of 110 (MACE: Cre = 113.10 μmol/L; NACE: Cre = 113.41 μmol/L; MACCE: Cre = 110.22 μmol/L). The RCS graph illustrates that the risk values for all three adverse events exhibit a significant upward trend prior to the inflection point, followed by a varying degree of downward trend afterward, demonstrating a classic J-shaped relationship (**Figure 2**).

According to the inflection point level (Cre = 110 μmol/L), we categorized the patient population for Cox regression analysis. In the adjusted model, MACE (*HR*: 1.155, 95%*CI*: 1.006~1.327), NACE (*HR*: 1.156, 95%*CI*: 1.004~1.330), and MACCE (*HR*: 1.161, 95%*CI*: 1.015~1.329) in the low creatinine level group (Creatinine ≤ 110 μmol/L) are significantly associated with creatinine levels (**Supplementary Table 2**).

### Subgroup analysis

**Figure 3:**
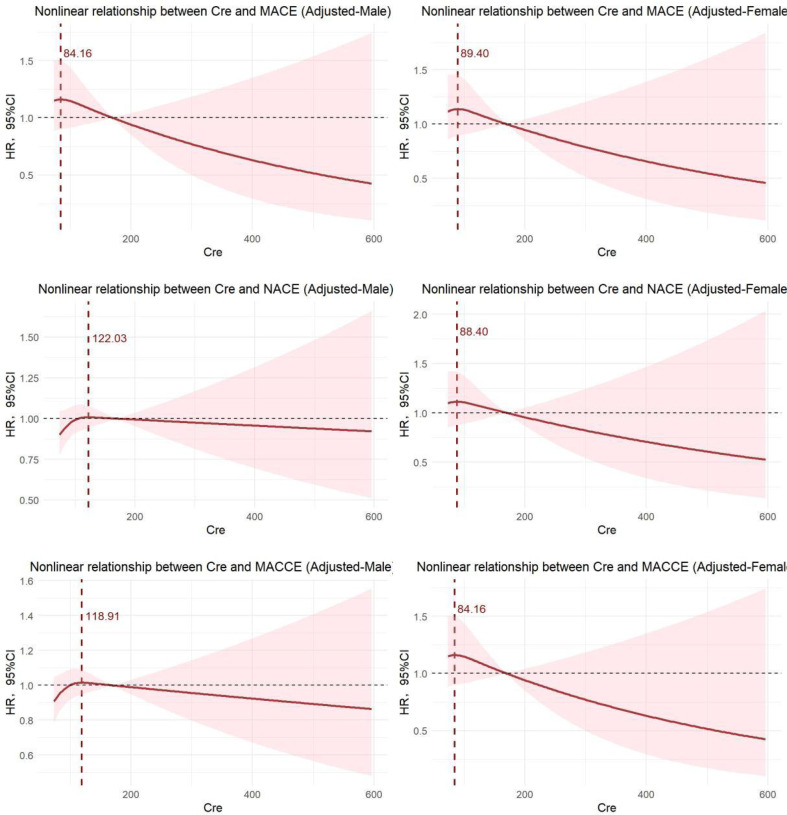
Nonlinear relationship between Cre and adverse events in different genders (Adjusted AMI type and diabetes).

We categorized the patients based on gender, revealing that the inflection point for MACE in the male population occurred at a Cre level of 84.16 μmol/L. In contrast, the inflection points for both NACE and MACE were approximately at Cre = 120 μmol/L. For the female population, the inflection point for all three types of adverse events was around Cre = 85 μmol/L. The RCS diagram illustrates that the risk values for the three types of adverse events exhibit a continuous downward trend to varying degrees after the inflection point, indicating a typical J-shaped correlation (**Figure 3**).

## Discussion

In this study, we explored the nonlinear correlation between admission serum creatinine (Cre) levels and adverse clinical events in patients with acute myocardial infarction (AMI) after percutaneous coronary intervention (PCI). Our findings reveal a significant J-shaped correlation between Cre levels and the risk of adverse events in this patient population. The J-shaped nonlinear association between serum Cre and adverse cardiovascular events observed in this study is not an isolated finding, but fits into a broader, well-validated paradigm of nonlinear risk relationships in cardiovascular disease, which challenges the traditional linear cognition of biomarker-prognosis associations (14, 15). Specifically, during the initial phase of low creatinine levels, the risk of adverse events increases rapidly with increasing creatinine. Once a certain threshold is reached, the variability in the risk of adverse events diminishes, and no significant difference is observed between the overall risk and the baseline risk. The inflection point for Cre levels is approximately 110 µmol/L. Furthermore, our study indicates that low levels of Cre may serve as a potential risk factor for adverse events in this patient group.

By definition, Cre levels rise sharply when the major excretory organ, the kidneys, is impaired. Numerous studies have demonstrated a strong association between Cre levels and AKI induced by major surgery (16, 17). In the present study, we enrolled 4, 541 patients who underwent PCI and identified a J-shaped relationship between Cre levels and adverse events, with an inflection point at 110 μmol/L. Notably, the HR increased rapidly before this inflection point; as Cre levels rose, the protective effect against adverse event risks gradually diminished and eventually transitioned into a risk factor. For each 1 μmol/L increase in Cre levels below 110 μmol/L, the risk of MACE increased by 15.5% (*HR*: 1.155, 95%*CI*: 1.006-1.327), the risk of NACE increased by 15.6% (*HR*: 1.156, 95%*CI*: 1.004-1.330), and the risk of MACCE increased by 16.1% (*HR*: 1.161, 95%*CI*: 1.015-1.329). These findings confirm that even Cre levels within the normal range are significantly associated with adverse events following PCI.

After PCI, Cre serves as the primary indicator of renal function, with elevated levels signifying a decline in glomerular filtration capacity (18). However, the occurrence of adverse events (MACE, NACE, MACCE) following PCI is frequently associated with renal hypoperfusion and metabolic disorders, exemplifying the intricate relationship within the heart-kidney interaction network (19). Numerous studies have demonstrated that an elevated baseline creatinine level can independently predict the risk of MACE after PCI (20, 21). Recent research indicates that certain dynamic changes in renal function indicators serve as superior postoperative predictors compared to baseline levels (22–25). In this study, we present for the evidence that the low level creatinine is closely correlated with the risk of adverse events in patients post-PCI. This J-shaped association holds significant clinical implications and offers crucial insights for refining individualized risk stratification strategies for patients after PCI, facilitating targeted interventions for early renal function protection, and establishing a foundation for future research aimed at elucidating the underlying mechanisms. The exact mechanism underlying this J-shaped association remains unclear; however, some existing studies may provide explanations: firstly, renal perfusion insufficiency, indicated by the dynamic increase in creatinine, can activate the renin-angiotensin-aldosterone system, leading to elevated blood pressure, increased myocardial oxygen consumption, and heightened risk of coronary artery ischemia (26). Additionally, fluctuations in creatinine may induce oxidative stress and inflammatory responses, exacerbating coronary endothelial dysfunction and promoting plaque instability (27, 28). Finally, even minor increases in baseline Cre within the normal range indicate occult reduction in renal functional reserve, which markedly increases susceptibility to contrast-induced acute kidney injury (CI-AKI) after PCI, and CI-AKI itself is a well-validated independent risk factor for adverse event (29), thus forming a critical mediating causal pathway linking low-range Cre elevation to adverse long-term clinical outcomes.

In addition, we determined that the inflection point of Cre associated with the risk of three types of adverse events after PCI was 110 μmol/L. It is generally accepted that a Cre level exceeding 110 μmol/L indicates the presence of early renal function impairment caused by acute renal perfusion insufficiency. Conversely, a Cre level below 110 μmol/L suggests recent renal function stability in the context of chronic renal insufficiency, or minor fluctuations in creatinine due to the self-regulation of renal blood flow following the procedure. However, this J-type correlation indicates that the risk of the aforementioned adverse events after PCI may increase even when the Cre level is below 110 μmol/L.

### Limitations

Despite providing evidence of the correlation between Cre levels and the risk of three types of adverse events (MACE, NACE, and MACCE) following PCI, several limitations of this study warrant clarification. Firstly, this study is a retrospective observational study, which cannot establish a definitive causal relationship between Cre levels and adverse events post-PCI; it can only reflect the correlation between these variables. Secondly, the data were sourced from an electronic medical record system, and certain potential confounding factors (such as the complexity of coronary artery disease and compliance with postoperative antiplatelet therapy) were not fully recorded, resulting in their exclusion from the multivariate correction model. Thirdly, the cohort consists of AMI patients post-PCI from this specific region, and their baseline characteristics (such as the extent of renal function impairment) are relatively homogenous. This may limit the generalizability of the findings to PCI patients in other regions. Therefore, the conclusions drawn regarding the influence of Cre levels on the risk of these three types of adverse events following PCI should be interpreted with caution and validated through multi-center prospective studies.

## Conclusion

In conclusion, this study reveals a J-type correlation between Cre levels and three types of adverse events (MACE, NACE, and MACCE) following PCI. Notably, low levels of Cre in patients post-PCI are significantly associated with the risk of these three types of adverse events, underscoring the importance of dynamically monitoring Cre levels and promptly identifying abnormal fluctuations in this clinical context. Furthermore, we have accurately determined that the inflection point of Cre related to adverse events post-PCI is 110 μmol/L, which can serve as a critical threshold for risk stratification and targeted intervention in patients after PCI. Future research should conduct multi-center, large-sample prospective cohort studies to further validate the findings of this study and explore the impact of individualized intervention strategies based on Cre levels on improving post-PCI prognosis.

## Data Availability

The raw data supporting the conclusions of this article will be made available by the authors, without undue reservation.
