# Supplementary material for: J-shaped relationship between creatinine levels and the risk of three major adverse events in patients after percutaneous coronary intervention
Source: Front Endocrinol (Lausanne). 2026 Apr 20;17:1832279. doi: 10.3389/fendo.2026.1832279 (PMC13136106; doi:10.3389/fendo.2026.1832279)
Supplement: Supplementary file 1 [file Table1.docx]

Supplementary Table 1. Baseline characteristics between adverse event groups in the study cohort

|  | MACE | |  | NACE | |  | MACCE | | VIF |
| --- | --- | --- | --- | --- | --- | --- | --- | --- | --- |
|  | *χ^2^*/*t*_MACE_ | *P*_MACE_ |  | *χ^2^*/*t*_NACE_ | *P*_NACE_ |  | *χ^2^*/*t*_MACCE_ | *P*_MACCE_ |  |
| Gender | 0.701 | 0.420 |  | 1.205 | 0.285 |  | 0.835 | 0.380 | 1.217 |
| Age | -0.972 | 0.331 |  | -1.195 | 0.232 |  | -1.292 | 0.197 | 1.175 |
| BMI | -1.006 | 0.315 |  | -1.528 | 0.127 |  | -0.899 | 0.369 | 1.080 |
| Hypertension | 4.330 | 0.038 |  | 4.782 | 0.030 |  | 5.390 | 0.021 | 1.013 |
| Hyperlipoidemia | 0.000 | 1.000 |  | 0.204 | 0.677 |  | 0.122 | 0.745 | 1.074 |
| Diabetes | 3.502 | 0.066 |  | 4.151 | 0.043 |  | 3.206 | 0.076 | 1.046 |
| Smoking | 0.000 | 1.000 |  | 0.090 | 0.755 |  | 0.013 | 0.931 | 1.039 |
| Drinking | 0.142 | 0.713 |  | 0.010 | 0.934 |  | 0.001 | 1.000 | 1.323 |

Supplementary Table 2. Cox regression to evaluate the relationship between Cre and adverse events in different groups through HR.

| Group | *β* | Wald | HR(95%CI) | *P* |
| --- | --- | --- | --- | --- |
| MACE |  |  |  |  |
| Cre≤110 | 0.0046 | 2.04 | 1.155(1.006, 1.327) | 0.0416 |
| Cre>110 | -0.0004 | -0.53 | 0.988(0.945, 1.033) | 0.5988 |
| NACE |  |  |  |  |
| Cre≤110 | 0.0047 | 2.02 | 1.156(1.004, 1.330) | 0.0436 |
| Cre>110 | -0.0004 | -0.48 | 0.989(0.945, 1.035) | 0.6293 |
| MACCE |  |  |  |  |
| Cre≤110 | 0.0048 | 2.17 | 1.161(1.015, 1.329) | 0.0301 |
| Cre>110 | -0.0004 | -0.50 | 0.989(0.946, 1.034) | 0.6148 |
