# Supplementary figures and images for: J-shaped relationship between creatinine levels and the risk of three major adverse events in patients after percutaneous coronary intervention
Source: Front Endocrinol (Lausanne). 2026 Apr 20;17:1832279. doi: 10.3389/fendo.2026.1832279 (PMC13136106; doi:10.3389/fendo.2026.1832279)

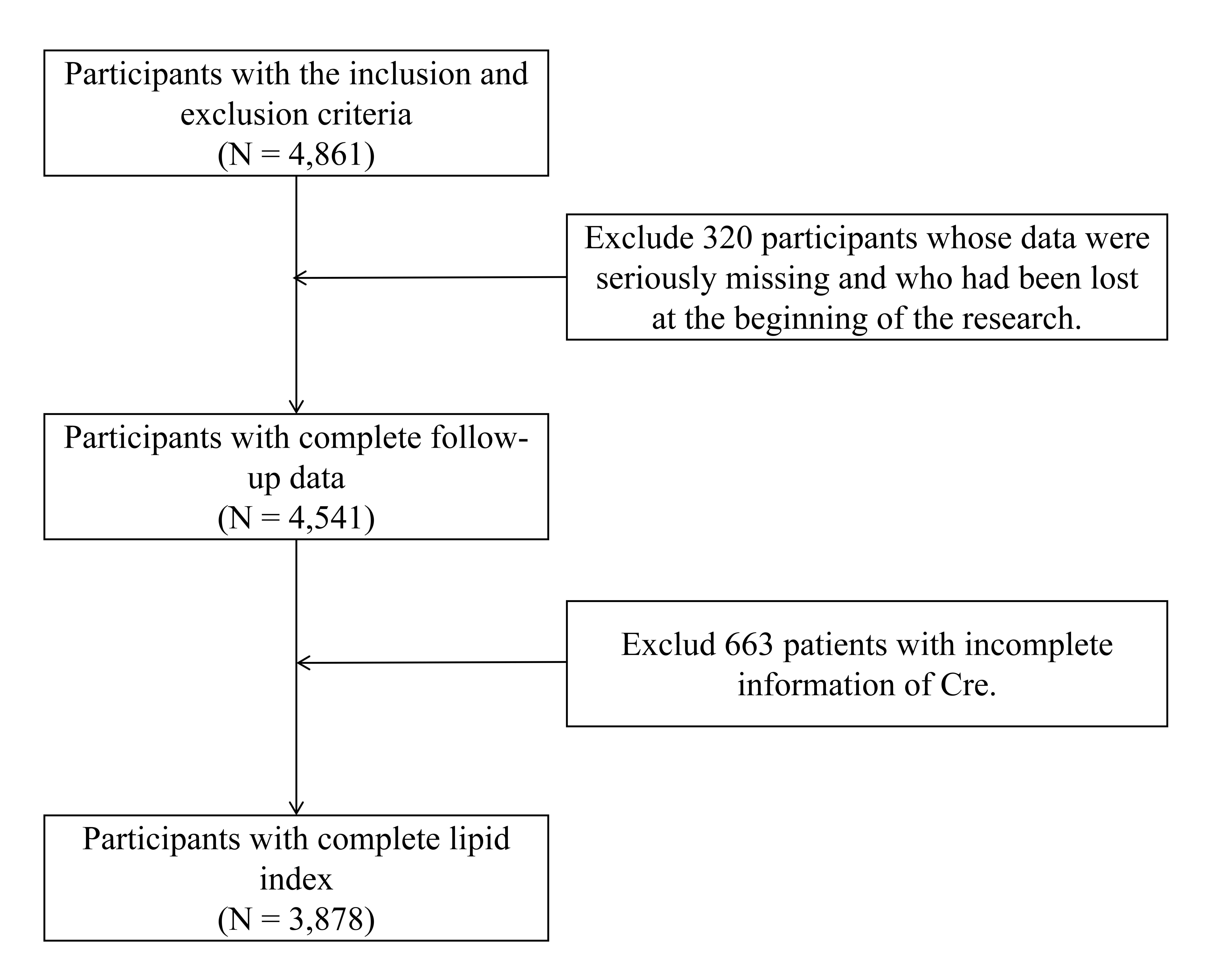

Supplement: Supplementary file 2 [file Image1.jpg]
